# Accumulation and Distribution Characteristics of Cd in the Soil-*Lilium* System and the Remediation Mechanism by Soil Amendments

**DOI:** 10.3390/plants14243798

**Published:** 2025-12-13

**Authors:** Yimin Zhou, Yulang Yan, Jiaxiang Wang, Yayuan Huang, Xinqi Wang, Bingyu Li, Ming Lei

**Affiliations:** 1 College of Environment and Ecology, Hunan Agricultural University, Changsha 410128, China; 2 Hunan Engineering and Technology Research Center for Irrigation Water Purification, Changsha 410128, China; 3 School of Metallurgy and Environment, Central South University, Changsha 410083, China; 4 Institute of Agricultural Environment and Ecology, Hunan Academy of Agricultural Sciences, Changsha 410128, China

**Keywords:** Soil-*Lilium* system, cadmium (Cd) accumulation, oyster shell powder, organic fertilizer, Cd reduction mechanism

## Abstract

This study investigated the accumulation and distribution of cadmium (Cd) in the Soil-*Lilium* system and researched the effects and mechanisms of applying oyster shell powder (OSP) and organic fertilizer (OF) on reducing Cd accumulation and enhancing *Lilium* yield. The results showed that the total Cd content in soils across different planting regions was below 0.3 mg·kg^−1^, while the Cd content in *Lilium* bulbs ranged from 0.44 mg·kg^−1^ to 1.35 mg·kg^−1^, indicating a consistent trend of Cd accumulation in *Lilium* bulbs. Cd contents were highest in the leaves and lowest in the bulbs, suggesting a strong translocation of Cd from the roots to the aerial parts. Both OSP and OF treatments improved *Lilium* growth and reduced Cd accumulation in the bulbs. OF significantly increased bulb yield by 62.5%, while OSP effectively reduced Cd content in the bulbs to 0.30 mg·kg^−1^, below the regulatory safety threshold. OSP mitigated Cd accumulation by decreasing the availability of Cd in the soil and by competing with Cd for root uptake via its abundant Ca^2+^ ions. OF reduced Cd accumulation in the bulb by enhancing Cd sequestration in the fibrous roots and promoting its translocation away from the bulb. This study provides new insights into Cd dynamics in the Soil-*Lilium* system and offers practical strategies for producing *Lilium* safely.

## 1. Introduction

*Lilium brownii var. viridulum* is a variety of *Lilium davidii Duch. ex Elwes* in the genus *Lilium* of *Liliaceae* family. It is rich in dietary fiber, protein, fat, amino acids, trace elements and vitamins, as well as various pharmacologically active components such as polysaccharides, alkaloids, steroidal saponins, flavonoids and phenolic compounds [1,2]. It has high edible, healthcare, and medicinal value, and is recognized as one of the 87 food-medicine homologous substances, as announced by the National Health Commission of China in 2011. The main organs of *Lilium* include succulent roots, bulbs, subterraneous stems, fibrous roots, aerial stems, leaves, and flowers with the main edible part being the bulb. The planting area of *Lilium* in China is widely distributed, with Lanzhou, Gansu Province; Shaoyang, Hunan Province; Yixing, Jiangsu Province and Huzhou, Zhejiang Province the major producing areas in China, with a cultivation area exceeding 20,000 hectares [3]. With the increase in work intensity and life pressure, a sub-health state has become more and more common, and the market share of *Lilium* has gradually risen [4]. At the same time, the problems of food safety and drug safety of *Lilium* have attracted attention at home and abroad.

In recent years, heavy metal contamination in the food chain has emerged as a significant public health concern. Cadmium (Cd), in particular, poses a severe threat due to its nephrotoxicity and neurotoxicity, which can damage the skeletal and cardiovascular systems and carry a carcinogenic risk [5,6]. Cd is characterized by its widespread dispersion in bedrock, high environmental mobility, and a propensity to enter the food chain [7]. As Cd has no known physiological function in plants, exposure to excess levels triggers a range of toxic effects. These include disruptions to key physiological processes such as photosynthesis, respiration, and transpiration [8]. Consequently, this leads to diminished photosynthetic capacity, resulting in stunted growth, oxidative stress, and impaired secondary metabolism [9]. Cd pollution in food and medicine homologous substances has been paid attention to by researchers. For example, Park et al. [10] found that the content range of Cd in *Poria cocos* was 0.001 mg·kg^−1^ to 0.085 mg·kg^−1^. Liu et al. [11] found that the content range of Cd in *Cassia obtusifolia* seeds, *Lycium barbarum*, *Platycodon grandiflorum* and *Taraxacum mongolicum* were 0.023 to 0.233 mg·kg^−1^, 0.32 to 0.061 mg·kg^−1^, 0.073 to 1.520 mg·kg^−1^ and 0.15 to 0.55 mg·kg^−1^ respectively. Compared with stem vegetables, solanaceous vegetables and leafy vegetables, bulb vegetables had higher Cd content [12]. Yang et al. [13] detected the Cd content in *Lilium* samples from Jiangxi and Hunan Provinces and found that the over-standard rate of Cd was 13.3%. Yang et al. [14] measured the average Cd content in Lanzhou *Lilium* as 0.324 mg·kg^−1^, exceeding the standard of 0.3 mg·kg^−1^. Huang et al. [15] analyzed the heavy metal content of *Lilium* samples collected from fields and purchased from pharmacies. The results showed that the content of Cd in *Lilium* was higher than other heavy metals, with its highest content reaching 1.45 mg·kg^−1^, exceeding the maximum allowable limit of 1.0 mg·kg^−1^ for Cd as specified in the Chinese Pharmacopoeia [15]. However, these studies only focused on the accumulation of Cd in the edible parts of *Lilium*, while the distribution and migration characteristics of Cd in other organs remained unclear. Therefore, it is significant to clarify the accumulation and distribution characteristics of Cd in Soil-*Lilium* system, which can further provide theoretical support for controlling Cd enrichment in *Lilium*.

At present, the prevention and control technology of heavy metal pollution in agricultural products has been widely concerned and studied. The passivation remediation method uses various amendments to immobilize heavy metals in soil through adsorption precipitation, complexation and other actions, reducing their mobility and bioavailability in the environment, which is a low-cost, easy-to-operate and environmentally friendly method for soil pollution remediation and prevention [16]. Various soil amendments, including shell powder, sepiolite, and organic fertilizers, have been explored for heavy metal remediation due to their distinct properties [17]. For example, shellfish shells, rich in calcium carbonate, can form a porous structure after calcination. This structure not only regulates soil pH and cultivates soil microorganisms but also facilitates the precipitation of heavy metal ions as carbonates, thus purifying the soil [18]. Similarly, clay minerals like sepiolite, with their large specific surface area, high CEC, and abundant negative surface charges, are also known for their high adsorption capacity for heavy metals and show great potential for soil passivation [19,20]. The application of organic fertilizers not only increases crop yield but also alters the physical and chemical properties of the soil, enhancing soil fertility [21]. Humic acid and fulvic acid in organic materials are the main active components that regulate the availability of heavy metals in the soil, generally believed to reduce the mobility and plant availability of heavy metals [22]. Some studies have found that soil Cd can form stable complexes with HA, reducing the migration ability of Cd [23]. Zhou et al. [24] discovered that biodegraded humic acid can decrease the proportion of exchangeable Cd in the total Cd of the soil. Research on the impact of heavy metal amendments on crops has largely focused on rice, corn, wheat, vegetables and other fields, with less research on medicinal and edible plants, and even less on their mechanisms.

To this end, the primary objectives of this study were to investigate the accumulation characteristics of Cd in the Soil-*Lilium* system across different regions, examine the distribution and migration patterns of Cd within various organs of *Lilium*, and identify commonalities in the accumulation, distribution and transfer of Cd within this system. Additionally, this study aimed to explore the effects of different soil amendments on inhibiting the enrichment of Cd in the edible parts of *Lilium*, as well as identify key environmental factors influencing these inhibitory effects. This will provide a theoretical basis for the safe production of food and for the control of Cd accumulation in *Lilium*.

## 2. Results

### 2.1. Cd Accumulation Characteristics of Soil-Lilium System

**Figure 1:**
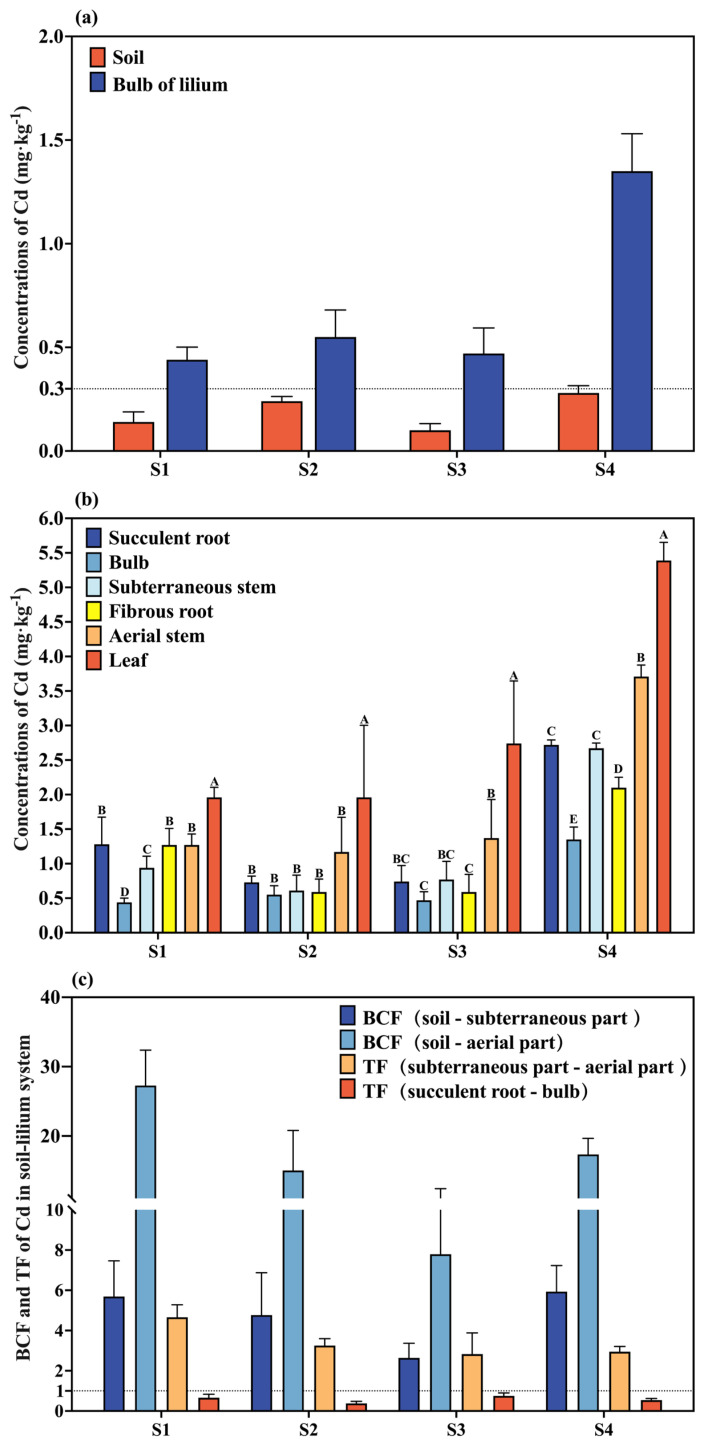
The Cd contents of the Soil-*Lilium* system in different regions. (**a**) Cd contents in soil and bulbs of *Lilium*; (**b**) Cd contents in each part of *Lilium*; (**c**) the bioaccumulation factor (BCF) and transfer factor (TF) of Cd in Soil-*Lilium* system. Data are mean SD (*n* = 3). Different letters indicate significant difference among treatments (*p* < 0.05).

Figure 1a shows the Cd content in soil and bulbs from four different areas of Longhui County, Hunan Province. The Cd content in the soil and *Lilium* bulbs from sampling site S1 was 0.14 mg·kg^−1^ and 0.44 mg·kg^−1^, respectively; at S2, 0.24 mg·kg^−1^ and 0.55 mg·kg^−1^; at S3, 0.10 mg·kg^−1^ and 0.47 mg·kg^−1^; and at S4, 0.28 mg·kg^−1^ and 1.35 mg·kg^−1^. The results indicate that the soil from all four sampling sites qualifies as Cd-clean soil (Cd < 0.3 mg·kg^−1^). However, Cd content in all *Lilium* bulbs exceeded the limit of 0.3 mg·kg^−1^ set by the “Green Trade Standard for Import and Export of Medicinal Plants and Preparations”, and the Cd content in the bulbs from site S4 exceeded the limit of 1.0 mg·kg^−1^ set by the Chinese Pharmacopoeia.

Figure 1b presents the Cd content in various parts of the *Lilium* plants from the four regions in Longhui County. At site S1, Cd concentrations were leaf (1.96 mg·kg^−1^) > succulent root (1.28 mg·kg^−1^) > aerial stem, fibrous root (1.27 mg·kg^−1^) > subterraneous stem (0.94 mg·kg^−1^) > bulb (0.44 mg·kg^−1^). At S2, they were leaf (1.96 mg·kg^−1^) > aerial stem (1.17 mg·kg^−1^) > succulent root (0.73 mg·kg^−1^) > subterraneous stem (0.61 mg·kg^−1^) > fibrous root (0.59 mg·kg^−1^) > bulb (0.55 mg·kg^−1^). At S3, they were leaf (2.74 mg·kg^−1^) > aerial stem (1.37 mg·kg^−1^) > subterraneous stem (0.77 mg·kg^−1^) > succulent root (0.75 mg·kg^−1^) > fibrous root (0.59 mg·kg^−1^) > bulb (0.47 mg·kg^−1^). At S4, they were leaf (5.39 mg·kg^−1^) > aerial stem (3.71 mg·kg^−1^) > succulent root (2.72 mg·kg^−1^) > subterraneous stem (2.67 mg·kg^−1^) > fibrous root (2.10 mg·kg^−1^) > bulb (1.35 mg·kg^−1^). The Cd content in the leaves of all *Lilium* plants was significantly higher than in other parts, while the Cd content in the bulbs was the lowest (*p* < 0.05). Except for site S1, the Cd content in the aerial parts of the plants was higher than in the subterraneous parts at all other sites.

Based on the Cd content in soil and various *Lilium* parts at maturity, the enrichment and transfer coefficients of Cd in the Soil-*Lilium* system were calculated. Figure 1c shows that the BCF of Cd in the subterraneous parts ranged from 2.64 to 5.93, while in the aerial parts, it ranged from 7.79 to 27.27. The TF from the subterraneous to the aerial parts ranged from 2.83 to 4.66, and the TF from the succulent root to the bulb ranged from 0.38 to 0.75. In all four regions, the BCF (soil-subterraneous part), BCF (soil-aerial part), and TF (subterraneous part-aerial part) were all greater than 1, while TF (succulent root-bulb) was less than 1. This indicates that *Lilium* has a strong ability to accumulate Cd from the soil, even in a low-pollution environment, where trace amounts of Cd in the soil can still lead to high Cd accumulation in the plants. The subterraneous parts also have a strong ability to transfer Cd upward, while the transfer of Cd from the succulent root to the bulb is relatively weak.

### 2.2. Effects of Amendments on Soil Physicochemical Properties and Cd Bioavailability

**Figure 2:**
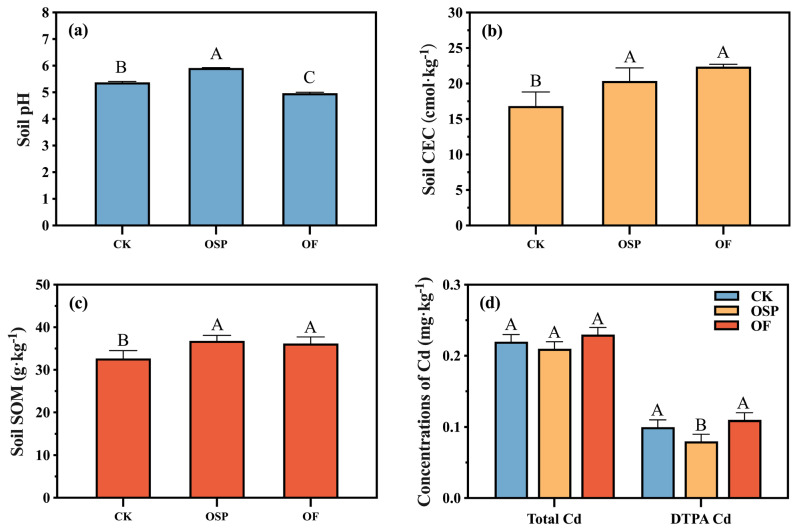
Effects of amendments on soil physicochemical properties and Cd bioavailability. (**a**) soil pH; (**b**) soil CEC; (**c**) soil SOM; (**d**) contents of soil total Cd and available Cd. Data are mean SD (*n* = 3). Different letters indicate significant difference among treatments (*p* < 0.05).

Figure 2a–c presents the changes in soil pH, CEC, and SOM content under different amendment treatments. pH is a key factor affecting the bioavailability of heavy metals in soil. The pH of the soil under the CK treatment was 5.38, which is classified as acidic. After applying OSP and OF, the soil pH showed different trends. Compared to CK, the soil pH increased by 0.54 under the OSP treatment and decreased by 0.41 under the OF treatment, indicating that OSP significantly increased the soil pH, while OF caused acidification of the topsoil (*p* < 0.05). Both amendments significantly increased the CEC by 3.51 and 3.89 cmol·kg^−1^ (*p* < 0.05) compared to CK. Additionally, the application of OSP and OF significantly affected the SOM, increasing it by 12.54% and 10.64%, respectively (*p* < 0.05).

The changes in total and bioavailable Cd in the soil under different treatments are shown in Figure 2d. Total Cd content in the soil was 0.22 mg·kg^−1^ under the CK treatment, 0.20 mg·kg^−1^ under OSP treatment, and 0.23 mg·kg^−1^ under OF treatment, all below the risk screening value for dry farming soil (0.3 mg·kg^−1^ at pH < 6.5) set by the Soil Environmental Quality Risk Control Standard for Agricultural Land (GB 15618-2018) [25]. Compared to CK, the total Cd content in the soil did not show significant changes with the application of OSP or OF, indicating no accumulation of Cd in the soil, and that the Cd content in these amendments did not cause additional Cd pollution. The bioavailable Cd content in the soil was significantly lower under OSP treatment (0.08 mg·kg^−1^) compared to CK (0.10 mg·kg^−1^, *p* < 0.05), a reduction of 20.00%, while the OF treatment increased bioavailable Cd by 10.00% (0.11 mg·kg^−1^), though this was not statistically significant (*p* > 0.05).

### 2.3. Effects of Amendments on the Growth of Lilium

**Figure 3:**
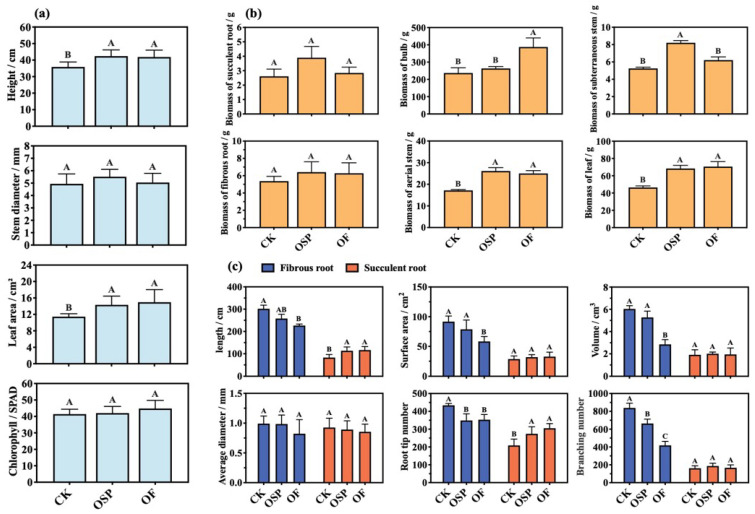
Effects of amendments on the growth of *Lilium*. (**a**) The growth index of *Lilium*; (**b**) the biomass of each part of *Lilium*; (**c**) changes of root morphology of *Lilium*. Data are mean SD (*n* = 3). Different letters indicate significant difference among treatments (*p* < 0.05).

Figure 3a shows the growth parameters of *Lilium* under OSP and OF treatments. Both amendments significantly promoted plant height and leaf area compared with CK (*p* < 0.05): plant height increased from 35.90 cm (CK) to 42.43 cm (OSP) and 41.88 cm (OF), while leaf area increased from 11.44 cm^2^ (CK) to 14.33 cm^2^ (OSP) and 14.97 cm^2^ (OF). Regarding stem diameter, the effects of both amendments were not statistically significant relative to CK (*p* > 0.05). Chlorophyll content increased by 1.52% (OSP) and 8.28% (OF) but without statistical significance relative to CK (*p* > 0.05). This indicates that both OSP and OF treatments promoted the growth of *Lilium*.

The changes in biomass across different parts of *Lilium* under the treatments are shown in Figure 3b. The bulb biomass under OSP and OF treatments was 263.70 g and 387.30 g, respectively, which represents an increase of 26.63 g (8.33%) and 150.23 g (62.5%) compared to CK (237.07 g). OF treatment showed a significant difference compared to the other two treatments. The results indicate that both OSP and OF treatments led to varying degrees of increase in the bulb yield at the maturation stage of *Lilium*, with OF treatment showing a significant yield improvement (*p* < 0.05). The biomass of the fibrous root in all treatments was higher than that of the succulent root. Under the OSP treatment, the biomass of the succulent root and fibrous root was 2.85 g and 6.29 g, respectively, while under the OF treatment, the biomass of these roots was 3.90 g and 6.41 g, respectively, both higher than CK treatment (2.61 g and 5.37 g), though not significantly different (*p* > 0.05). The biomass of the aerial stem in all treatments was higher than that of the subterraneous stem. For OSP treatment, both subterraneous stem biomass and aerial stem biomass were significantly higher than those of CK (*p* < 0.05). In contrast, OF treatment only induced a significant increase in aerial stem biomass compared to CK, while the subterraneous stem biomass showed no statistical difference from CK despite a substantial increment. The leaf biomass under OSP and OF treatments was 70.64 g and 68.37 g, respectively, both significantly higher than CK (46.45 g, *p* < 0.05). The results indicate that both soil amendments promoted the growth of various parts of the *Lilium*, with the increase in succulent root and subterraneous stem biomass being greater under the OSP treatment than under the OF treatment.

Changes in root morphology under different treatments are shown in Figure 3c. In the CK group, the length, surface area, volume, average diameter, root tip number and branching number of the fibrous root were 301.82 cm, 91.68 cm^2^, 6.04 cm^3^, 0.99 mm, 434 and 839, respectively. Under the OSP treatment, these indicators were 85.38%, 85.90%, 87.38%, 99.31%, 80.41% and 79.14% of CK, while under the OF treatment, these indicators were 75.17%, 63.72%, 47.23%, 82.91%, 81.34% and 49.94% of CK. Compared with CK, all indicators of the fibrous root under OSP and OF treatments decreased, with the root tip number and branching number significantly lower under the OSP treatment and the length, surface area, volume, root tip number and branching number significantly lower under the OF treatment (*p* < 0.05). For the succulent root, the length, surface area, volume, average diameter, root tip number and branching number in the CK group were lower than those of the fibrous root at 83.29 cm, 28.69 cm^2^, 1.91 cm^3^, 0.93 mm, 209 and 162, respectively. Under the OSP treatment, these indicators were 136.26%, 111.36%, 105.29%, 96.20%, 131.10%, and 115.43% of CK, while under the OF treatment, they were 140.43%, 114.35%, 101.93%, 92.18%, 145.93%, and 103.09% of CK. Compared with CK, all indicators of the succulent root under OSP and OF treatments increased, except for the average diameter. The length and root tip number showed significant increases. These results indicate that OSP and OF treatments had a greater impact on the root morphology of the fibrous root than the succulent root, with the OF treatment having a greater influence than the OSP treatment.

### 2.4. Effects of Amendments on the Cd Accumulation Characteristics of Lilium

**Figure 4:**
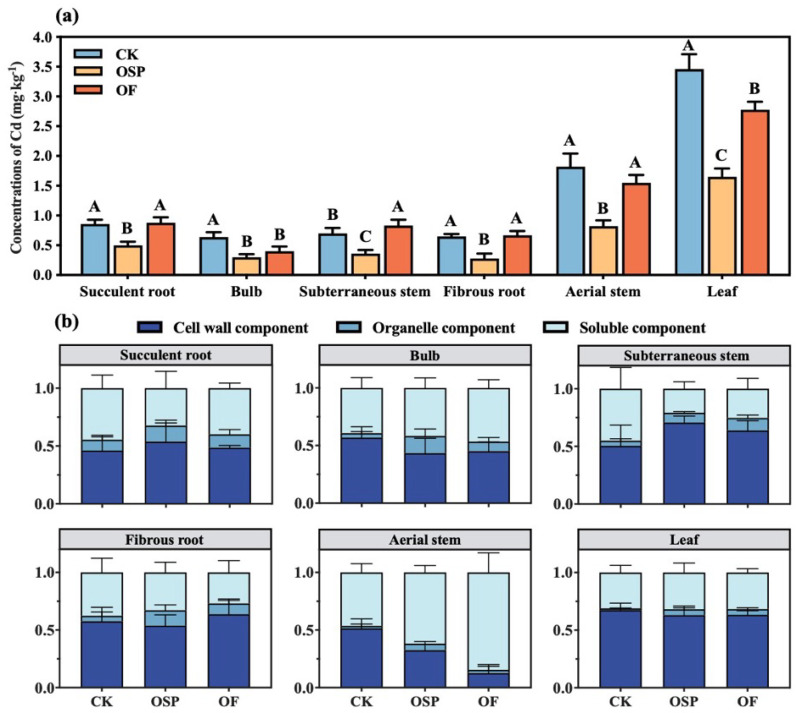
Effects of amendments on the Cd accumulation of *Lilium*. (**a**) Cd contents in different parts of *Lilium*; (**b**) subcellular distribution of Cd in different parts of *Lilium*. Data are mean SD (*n* = 3). Different letters indicate significant difference among treatments (*p* < 0.05).

The Cd content in different parts of *Lilium* under various treatments is shown in Figure 4a. There was a significant variation in Cd accumulation across different *Lilium* organs, with the Cd content in the succulent root, bulb, subterraneous stem, fibrous root, aerial stem and leaves ranging from 0.50 to 0.88 mg·kg^−1^, 0.30 to 0.64 mg·kg^−1^, 0.36 to 0.83 mg·kg^−1^, 0.28 to 0.67 mg·kg^−1^, 0.82 to 1.82 mg·kg^−1^, and 1.65 to 3.46 mg·kg^−1^, respectively. In all treatments, the Cd content in the bulb was lower than the limit value (1.0 mg·kg^−1^) specified in the 2020 edition of the Chinese Pharmacopoeia. As shown in Figure 4a, Cd primarily accumulated in the leaves, with the Cd content in the aerial parts of *Lilium* being significantly higher than that in the subterraneous parts (*p* < 0.05). Across different treatments, the Cd content distribution in the various parts of *Lilium* followed a consistent pattern: leaves > aerial stem > succulent root > subterraneous stem > fibrous root > bulb. Under the OSP treatment, the Cd content in all parts of *Lilium* significantly decreased compared to CK, with reductions in the succulent root, bulb, subterraneous stem, fibrous root, aerial stem and leaves of 41.86%, 50.00%, 48.57%, 56.92%, 54.95% and 52.59%, respectively (*p* < 0.05). Under the OF treatment, the Cd content in the succulent root, subterraneous stem and fibrous root increased compared to CK, with the subterraneous stem showing a significant increase of 18.57%. However, the Cd content in the bulb, aerial stem and leaves decreased compared to CK, with the bulb and leaves showing significant reductions of 37.50% and 20.11%, respectively. Except for the bulb, the Cd content in other parts of *Lilium* was significantly lower under the OSP treatment compared to the OF treatment (*p* < 0.05). The results suggest that the OSP treatment significantly inhibited Cd uptake by *Lilium* roots from the soil, while the OF treatment promoted Cd absorption by the roots, though without significant impact. Both OSP and OF treatments significantly reduced the Cd content in the edible part (bulb) of *Lilium* (*p* < 0.05). Under the OSP treatment, the Cd content in the bulb was 0.30 mg·kg^−1^, meeting the limit value (0.3 mg·kg^−1^) of the “Green Trade Standard for Import and Export of Medicinal Plants and Preparations”, and the OSP treatment was significantly more effective than the OF treatment in reducing Cd content in other parts of *Lilium* (*p* < 0.05).

To explore the effects of different soil amendments on Cd translocation and transport between various organs of *Lilium*, this study analyzed the subcellular distribution of Cd in *Lilium* organs at the maturation stage, as shown in Figure 4b. The results indicate that under CK treatment, the subcellular distribution of Cd in various *Lilium* organs was consistent, with Cd mainly concentrated in the cell wall and soluble components. Both OF and OSP treatments increased the Cd content in the cell wall components of the succulent root and subterraneous stem while reducing the Cd content in the cell wall components of the bulb and aerial stem. There was no significant effect on the subcellular components of the fibrous root and leaves. After the application of amendments, the soluble Cd in the bulb and aerial stem increased.

### 2.5. Principal Component Analysis and Pearson Correlation Coefficient

**Figure 5:**
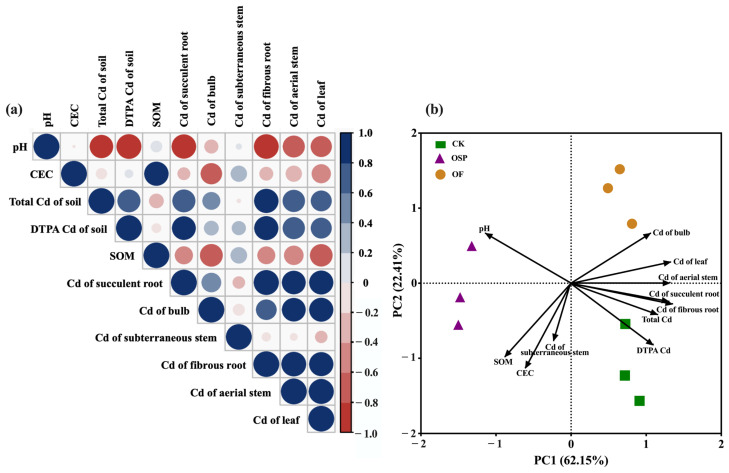
Pearson correlation (**a**) and principal component analysis (**b**) between soil factors and Cd contents under different treatments.

To explore the correlation between various soil factors and the Cd content in different organs of *Lilium*, Pearson correlation analysis was conducted, and the results are shown in Figure 5a. Soil pH exhibited a highly significant negative correlation with the Cd content in the soil, fibrous root and succulent root (*p* < 0.01) and a significant negative correlation with the Cd content in the aerial stem (*p* < 0.05). This indicates that adjusting soil pH is an effective method to alter the form of Cd in the soil and the uptake and transport of Cd in *Lilium* plants. The available Cd content in the soil showed a strong positive correlation with the Cd content in the succulent root, fibrous root and aerial stem (r > 0.70), suggesting that available Cd in the soil might be the primary source of Cd absorption by *Lilium* roots and Cd transport to the aerial stem. Soil CEC was highly positively correlated with SOM content (*p* < 0.01), and both were significantly negatively correlated with the Cd content in the bulb (*p* < 0.05), indicating that increased soil CEC and SOM content may play a key role in reducing Cd accumulation in the bulb. Additionally, SOM content was significantly negatively correlated with the Cd content in the leaves (*p* < 0.05). There were significant positive correlations between the Cd content in the succulent root, fibrous root, aerial stem and leaves (*p* < 0.01). The Cd content in the bulb was highly significantly positively correlated with the Cd content in the aerial stem and leaves (*p* < 0.01), while the subterraneous stem showed no significant correlation with the Cd content in other parts of *Lilium* or soil factors.

Figure 5b displays the principal component analysis (PCA) of all influencing factors under different treatments. As shown, PC1 and PC2 together explained 91.16% of the total variance, with contributions of 67.04% and 24.12%, respectively. The Cd content in the roots and aerial stem contributed significantly to PC1, while soil CEC contributed largely to PC2. There was a significant negative correlation between soil pH and PC1. The sample points within each treatment group showed clustering, indicating high similarity and good reproducibility within the groups. The first principal component distinctly separated the OSP group from the other two treatment groups, while the sample points from the CK and OF groups were clearly separated along the second principal component. The CK treatment group was distributed negatively on the PC2 axis, while the OF treatment group was distributed positively, indicating significant differences between the three treatment groups, with greater differences between the OSP group and the other two groups than between the CK and OF groups. Regarding the relationships between the variables, the available Cd content in the soil was positively correlated with the Cd content in various *Lilium* organs and negatively correlated with soil pH, consistent with the conclusions from the Pearson correlation analysis. As for the relationship between the variables and sample points, soil pH, CEC and SOM content were more strongly correlated with the OSP treatment.

## 3. Discussion

### 3.1. Accumulation, Distribution and Migration of Cd in Soil-Lilium System

The results showed that the total Cd content in the soil from the four sampling sites ranged from 0.10 mg·kg^−1^ to 0.28 mg·kg^−1^ (Figure 1a), all of which were below the risk screening value (0.3 mg·kg^−1^) set by the Risk Control Standards for Soil Pollution of Agricultural Land (GB15618-2018) [25]. This indicates that the soil in the four *Lilium* planting areas involved in the study is considered clean. However, the total Cd content in the *Lilium* bulbs from the sampling sites ranged from 0.44 mg·kg^−1^ to 1.35 mg·kg^−1^ (Figure 1b), which was higher than the corresponding soil Cd content as well as the Cd limit of 0.3 mg·kg^−1^ set by the Green Trade Standards of Importing & Exporting Medicinal Plants & Preparations. In some cases, the maximum value even exceeded the Cd limit of 1.0 mg·kg^−1^ in the Chinese Pharmacopoeia, indicating a common tendency for *Lilium* bulbs to accumulate Cd from the soil. Some studies have also found excessive levels of heavy metals in bulbs of *Lilium* [15,26,27].

The distribution of Cd content across different *Lilium* organs followed the general pattern of leaves, aerial stem and fibrous root > subterraneous stem, succulent root and bulb (Figure 1b). The leaves had the highest Cd content, while the bulb had the lowest, suggesting that the fibrous root has a strong ability to accumulate Cd, and the aboveground parts of the *Lilium* plant accumulate more Cd than the belowground parts. The distribution and accumulation patterns of Cd across *Lilium* plants from different regions showed similarity. Previous studies have also indicated a similar trend in tuberous plants like potatoes, where heavy metal accumulation followed the pattern: leaves > roots ≈ stems > tubers, which parallels the Cd accumulation pattern in *Lilium* [28].

The bioaccumulation factors (BCFs) of the above- and belowground parts of *Lilium* for soil Cd content across the four regions were all greater than 1, with the BCF of the aboveground parts reaching as high as 7.79–27.27 (Figure 1c). Additionally, the translocation factor (TF) from belowground to aboveground parts of *Lilium* was also greater than 1, indicating a strong ability of *Lilium* plants to absorb Cd from the soil and transport it upwards in the Soil-*Lilium* system. Different plant species differ in their ability to absorb, transport and accumulate Cd, and Cd is transported from the soil to roots through different transporters that are used for the uptake of essential plant nutrients [29]. Therefore, it is speculated that the *Lilium* absorbs and accumulates Cd in the process of taking nutrients into the soil. However, the TF from fibrous root to bulb was less than 1 in all four regions, suggesting that the transfer of Cd from the fibrous root to the bulb is weak. This limited translocation may be a key tolerance mechanism in *Lilium*. A well-established strategy for heavy metal detoxification in plants is the sequestration of metals into cellular vacuoles, which isolates them from vital metabolic processes and protects growth and reproductive functions [30]. It is plausible that *Lilium* employs this mechanism, preferentially sequestering Cd in the vacuoles of root cells to restrict its transport to the bulb, a crucial reproductive organ.

### 3.2. Mechanisms of Cd Immobilization and Translocation Regulation in Lilium

Both soil amendments effectively reduced Cd accumulation in *Lilium* bulbs, albeit through distinct mechanisms (Figure 4a). Oyster shell powder (OSP) primarily acted by immobilizing Cd in the soil, thereby reducing its bioavailability and uptake. In contrast, organic fertilizer (OF) appeared to regulate the internal translocation of Cd within the plant after uptake, redirecting it away from the edible bulb.

The superior performance of OSP in reducing bulb Cd content can be attributed to its profound impact on soil chemistry. Firstly, OSP is rich in CaCO_3_, which significantly raised the soil pH (Figure 2a). This increase in pH promotes the adsorption and precipitation of Cd, thereby decreasing its bioavailability and mobility in the soil [31,32]. Turan [33] found that applying eggshell powder to the soil significantly reduced the Pb content in plant roots and stems. Secondly, OSP application significantly increased both soil CEC and SOM (Figure 2b,c). The inorganic carbon from OSP dissolution provides a substrate for soil autotrophic microorganisms. These microbes assimilate the carbon into new biomass, which, upon their death and turnover, becomes incorporated into stable SOM [18]. The raised CEC, enhancing the adsorption of heavy metals by soil colloids, and increased SOM, promoting the formation of organic-bound Cd [34]. Collectively, these changes led to a significant reduction in the amount of available Cd in the soil (Figure 2d), fundamentally limiting the plant’s Cd uptake.

Furthermore, Cd, as a non-essential element, lacks specific transporters and pathways within plants, the high concentration of metal cations (e.g., Ca^2+^, Mg^2+^) released from OSP likely induced competitive inhibition at the root membrane transporters, decreasing Cd absorption [30,35]. The added Ca^2+^ is also a crucial nutrient that enhances the synthesis and thickness of root cell walls [36]. Our results confirmed this, showing a significant increase in Cd immobilization within the succulent root and subterraneous stems cell walls under OSP treatment, which acted as a final barrier, further restricting Cd transfer to the bulbs (Figure 4b). Beyond Cd mitigation, the calcium and silicon compounds in OSP are known to improve plant structural integrity and stress resistance [37]. This contributes to improved overall plant vigor and quality, making OSP a dual-function amendment for both safety and yield enhancement.

In contrast to OSP, OF lowered soil pH, which would typically increase Cd bioavailability (Figure 2a,d). However, OF still achieved a significant reduction in bulb Cd content (Figure 4a) while exhibiting a greater biomass effect (Figure 3a,b). This indicates that OF’s primary mechanism is not soil immobilization but the regulation of Cd translocation within the *Lilium* plant itself, coupled with its nutritional benefits. Firstly, the organic matter, calcium, and magnesium in OF provide the building blocks for synthesizing cell wall components in the fibrous roots and subterranean stems [38]. The low-methylated pectin in these cell walls, rich in carboxyl groups, can bind Cd^2+^ through electrostatic interactions, forming a primary barrier that immobilizes a majority of the absorbed Cd in the roots [39]. Concurrently, OF induced adaptive changes in root morphology, characterized by a significant reduction in the number of root tips and bifurcations (Figure 3c). This morphological adaptation weakens the plant’s overall capacity for Cd absorption, thereby decreasing the total Cd load entering the plant [40]. Secondly, for the Cd that bypasses the root defense and enters the bulbs (the reproductive organs), OF promotes its sequestration into the soluble fraction, primarily the vacuole. This process is likely mediated by the synergistic action of vacuolar proton pumps (H^+^-ATPase) and metal/H^+^ antiporters, which actively transport Cd^2+^ into the vacuole for detoxification [41]. Our data showed that under OF treatment, the proportion of Cd in the soluble fraction of the bulb increased, supporting this compartmentalization strategy (Figure 4b). Thirdly, driven by a strong reproductive protection strategy, the bulb sequesters Cd in the vacuole to protect its metabolic functions. This vacuolar Cd can be readily remobilized and translocated via vascular tissues to the leaves (non-reproductive, sink organs) [42]. This is evidenced by our findings that while total Cd in the bulb decreased, the proportion of soluble Cd in the aerial stems and leaves significantly increased under OF treatment (Figure 4a). Ultimately, this mechanism achieves a targeted redistribution of Cd, safeguarding the reproductive bulb by diverting the toxicant to less critical aerial parts. Crucially, the biomass-increasing effect of OF stems from its rich composition of decomposed animal manure, rapeseed meal, and wheat bran. These components provide a comprehensive supply of essential macro- and micronutrients, directly fueling plant growth and development [43]. This nutritional benefit explains why OF demonstrated a greater effect on increasing yield compared to OSP, making it a highly effective amendment for enhancing both the safety and economic value of the crop.

## 4. Materials and Methods

### 4.1. Field Investigation and Sample Collection

**Figure 6:**
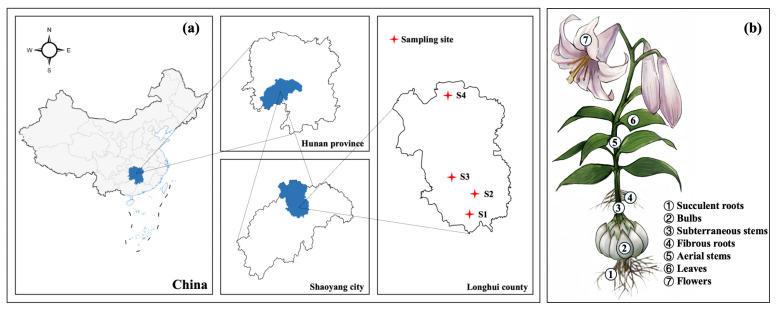
The sampling sites of Longhui County, Hunan Province, China (**a**) and the morphology of *Lilium* (**b**).

To assess the accumulation and distribution characteristics of Cd in the Soil-*Lilium* system, a field investigation was conducted in August 2021 in Longhui County, Hunan Province, a primary production area for *Lilium brownii var. viridulum*. Four representative planting areas were selected as sampling sites (Figure 6a). While all sampling sites shared a uniform yellow-brown soil type, their detailed physicochemical properties varied and are provided in Table S1. Critically, total Cd concentrations at all sites were below the national safety limit of 0.3 mg·kg^−1^.

In each planting area, three fields were randomly selected using a grid-based approach to ensure uniform coverage and avoid bias. From each field, a composite soil sample (0–20 cm) and a composite sample of mature whole *Lilium* plants were collected using the five-point sampling method, resulting in a total of three independent composite samples per planting area.

### 4.2. Selection of Soil Amendments and Field Experiment

This study employed a two-stage experimental approach to identify and validate effective soil amendments for reducing Cd accumulation in *Lilium*.

#### 4.2.1. Preliminary Screening of Amendments

As showed in SI, a preliminary screening experiment was conducted to evaluate the efficacy and cost-effectiveness of four soil amendments: Oyster Shell Powder, Sepiolite, Organic Fertilizer A and Organic Fertilizer B. The detailed composition and nutritional characteristics of these amendments are provided in Table S2. Each amendment was tested at two application rates (4500 and 9000 kg·ha^−1^) with three replicates.

The results of this screening showed that all four amendments reduced the Cd concentration in *Lilium* bulbs compared to the control (Figure S1). Notably, Oyster Shell Powder and Organic Fertilizer A demonstrated the high Cd immobilization efficiency, reducing bulb Cd by 70.9% and 71.7%, respectively, at the 4500 kg·ha^−1^ rate (Figure S1). Increasing the application rate to 9000 kg·ha^−1^ did not yield a proportionally greater reduction in Cd. Based on a comprehensive evaluation of Cd immobilization efficiency and application cost, Oyster Shell Powder and Organic Fertilizer A (hereafter referred to as Organic Fertilizer) at the rate of 4500 kg·ha^−1^ were selected as the optimal treatments for the subsequent field validation.

#### 4.2.2. Field Validation Experiment

A field experiment was established in September 2021 at a *Lilium* planting base in Longhui County to validate the effectiveness of the selected amendments and elucidate the associated mechanisms causing the reduced uptake of Cd by *Lilium*. The soil used for the experiment had the following characteristics: pH 5.38, cation exchange capacity (CEC) 16.84 cmol·kg^−1^, soil organic matter (SOM) 32.70 g·kg^−1^, total Cd content 0.22 mg·kg^−1^, and available Cd content 0.10 mg·kg^−1^. The tested *Lilium* variety was *Lilium brownii var. viridulum*. The experiment consisted of three treatments: a control (CK), Oyster Shell Powder (OSP), and Organic Fertilizer (OF), each with three replicates in a randomized complete block design for a total of nine plots (20 m^2^ each). OSP and OF was applied at the optimal rate of 4500 kg·ha^−1^ and incorporated into the top 20 cm of soil. The CK group received no amendment. After a 20-day equilibration period, *Lilium* bulbs were sown. All other field management practices followed local standards.

At the *Lilium* maturity stage in August 2022, agronomic traits and physiological parameters were measured in situ in each plot prior to destructive sampling. To avoid subjective selection, ten *Lilium* plants were randomly selected from within the plot by walking along a ‘W’-shaped transect and selecting the nearest plant at regular intervals. For these selected plants, plant height and leaf area were measured using a tape measure, while stem diameter was measured using a vernier caliper. Relative chlorophyll content (SPAD value) was determined on functional leaves (fully expanded, disease-free, and from similar canopy positions) per plant using a portable chlorophyll meter SPAD-502 Plus (Konica Minolta, Osaka, Japan). Subsequently, soil samples from the 0–20 cm and whole *Lilium* plants were collected from each plot using the five-point sampling method.

### 4.3. Sample Preparation and Analysis

#### 4.3.1. Soil Sample

All collected soil samples were air-dried, ground and passed sequentially through a series of sieves to meet the requirements of different analyses. The fraction passing a 10-mesh (2 mm) sieve was used for total N, *p*, and K analysis. The fraction passing a 20-mesh (0.85 mm) sieve was used for pH, CEC, and available Cd measurements. The fraction passing a 100-mesh (0.15 mm) sieve was used for total Cd and organic matter analysis. All processed samples were stored in sealed plastic bags until analysis.

Soil physicochemical properties were determined according to the methods described by Lu [44]. Total N concentration was measured by the semi-micro Kjeldahl digestion method. Total *p* was determined by the molybdenum-antimony colorimetric method, and total K was measured by flame photometry. Soil pH was measured in a 1:2.5 (*w*/*v*) soil-to-water suspension using a pH meter (Seven Compact S220, Mettler Toledo, Zurich, Switzerland). SOM was determined by the potassium dichromate oxidation-titration method. CEC was determined using the cobalt hexamine trichloride extraction-spectrophotometry method.

The total and available Cd content in the soil was conducted following the protocol of Zhou et al. [45]. Typically, total Cd was extracted using the aqua regia digestion method, and available Cd was extracted using the diethylenetriaminepentaacetic acid solution (DTPA). The Cd concentrations in the digestion and extraction solutions were quantified using an inductively coupled plasma mass spectrometer (ICP-MS, NexION 300X, PerkinElmer, Waltham, MA, USA). For quality assurance and quality control purposes, blanks and standard soil reference material (GBW07406, GSS-6) were digested along with the unknown samples.

#### 4.3.2. *Lilium* Sample

All *Lilium* plants were washed thoroughly with tap water followed by deionized water. Plants were then divided into six parts: succulent root, bulb, subterraneous stem, fibrous root, aerial stem, and leaf (Figure 6b). Flowers were not included as sampling occurred post-flowering. For each part, a subsample of fresh tissue (~20 g) was stored at -80 °C for subcellular Cd analysis. The remaining tissue was oven-dried at 105 °C for 1 h to deactivate enzymes, then at 65 °C to a constant weight. The dried samples were ground using a stainless-steel grinder and stored in sealed plastic bags for chemical analysis of total Cd concentration.

In parallel, for root morphology analysis, a separate subsample of fresh fibrous and succulent roots was carefully washed with deionized water to remove all adhering soil. Immediately after washing, these fresh roots were patted dry and scanned at 600 dpi using a scanner Epson Expression 1680 (Seiko Epson Corp, Tokyo, Japan). The resulting scanned images were then analyzed using the WinRHIZO root analysis system (Regent Instruments Inc., Quebec, QC, Canada) to quantify morphological parameters such as total length, surface area, and volume.

Subcellular fractions of the *Lilium* tissues were separated using differential centrifugation, following the protocol described by Feng et al. [46]. This procedure partitioned the tissue into three main components: the cell wall component (consisting of cell walls and cell wall debris), the organelle component (including chloroplasts, mitochondria, and nuclei), and the soluble component (the cytosol and vacuolar contents). Samples from each fraction were digested with HNO_3_, and the Cd content in digestion solutions was measured as standing for their content in the cell wall, organelle, and soluble component.

To determine the total Cd content, dried and ground *Lilium* plant samples were digested with a mixture of concentrated HNO_3_ and HClO_4_ (4:1, *v*/*v*) [45]. The Cd concentrations in the final digestion solutions were measured using ICP-MS NexION 300X (PerkinElmer, Waltham, MA, USA). Blanks and standard plant reference material (GBW10049, GSB-27) were digested along with the unknown samples and used for the QA/QC program.

### 4.4. Parameter Calculation

The bioconcentration factor (BCF) represents the plant’s ability to accumulate heavy metals from the soil [47]. In this study, the *Lilium* plant was divided into underground and aboveground parts, and the BCF was calculated as the ratio of the Cd content in the *Lilium* plant to the Cd content in the soil. BCF was calculated as follows:

C_underground_ = (C_1_·m_1_ + C_2_·m_2_ + C_3_·m_3_ + C_4_·m_4_)/(m_1_ + m_2_+ m_3_ + m_4_) (1)

C_aboveground_ = (C_5_·m_5_ + C_6_·m_6_)/(m_5_ + m_6_) (2)

BCF = C_underground_/C_soil_ or BCF = C_aboveground_/C_soil_ (3)

where C_1_, C_2_, C_3_, C_4_, C_5_ and C_6_ represent the Cd content in the succulent root, bulb, subterraneous stem, fibrous root, aerial stem, and leaf, respectively. The m_1_, m_2_, m_3_, m_4_, m_5_, and m_6_ represent the dry weight of the succulent root, bulb, subterraneous stem, fibrous root, aerial stem, and leaf, respectively. C_underground_ represents the Cd content in the underground parts of the *Lilium* plant, C_aboveground_ represents the Cd content in the aboveground parts, and C_soil_ represents the Cd content in the soil.

The translocation factor (TF) represents the ability of Cd to move within the *Lilium* plant, and it was calculated as the ratio of Cd content in the subsequent part to the Cd content in the preceding part [48]. The translocation from the underground parts to the aboveground parts and from the succulent root to the bulb was specifically calculated. TF was calculated as follows:

TF = C_after_/C_former_ (4)

where C_former_ represents the Cd content in the preceding part (underground part/succulent root) and C_after_ represents the Cd content in the subsequent part (aboveground part/bulb).

### 4.5. Statistical Analysis

Data were organized using Microsoft Excel 365, statistical analyses were conducted using SPSS Statistics 26.0, and graphs were created using GraphPad Prism 9.0 and R Studio 2024.12.1+563. One-way analysis of variance (ANOVA) and LSD multiple comparison tests were used to determine significant differences, with *p* < 0.05 considered statistically significant. Pearson correlation analysis was conducted to assess relationships between variables, and principal component analysis (PCA) was performed using R Studio.

## 5. Conclusions

This study investigated the accumulation and distribution of Cd in the Soil-*Lilium* system and evaluated the effects of OSP and OF as soil amendments. The results indicated that while total Cd in the soil was below regulatory thresholds, *Lilium* bulbs exhibited significant accumulation, posing a potential food safety risk. The application of OSP effectively reduced the Cd content in bulbs to below the safety threshold, primarily by immobilizing Cd in the soil and by competing with Cd for root uptake via its abundant Ca^2+^ ions. Concurrently, the application of OF increased the bulb yield by 62.5% and reduced Cd accumulation in the bulb by sequestering Cd in the fibrous roots and promoting its translocation to other plant parts. These findings provide practical strategies for the safe cultivation of *Lilium*, emphasizing the potential of environmentally friendly amendments to enhance both plant growth and food safety.

## Data Availability

The datasets generated and/or analyzed during the current study are available from the corresponding authors upon reasonable request. The data are not publicly available due to the ongoing nature of the research project and restrictions imposed by the associated research agreements.

## Supplementary Materials

The following supporting information can be downloaded at: https://www.mdpi.com/article/10.3390/plants14243798/s1, Table S1. Soil physicochemical properties of sampling sites; Table S2. Detailed composition and nutritional characteristics of amendments; Figure S1. Cd content in bulbs of *Lilium* under different soil amendment treatments.
