# Supplementary material for: Accumulation and Distribution Characteristics of Cd in the Soil-Lilium System and the Remediation Mechanism by Soil Amendments
Source: Plants (Basel). 2025 Dec 13;14(24):3798. doi: 10.3390/plants14243798 (PMC12737069; doi:10.3390/plants14243798)
Supplement: Supplementary file 1 [file plants-14-03798-s001.zip › plants-4016226-supplementary.pdf]

Supplementary Information

Accumulation and Distribution Characteristics of Cd in the Soil-*Lilium* System and the Remediation Mechanism by Soil Amendments

Yimin Zhou [1,2], Yulang Yan [1,2], Jiaxiang Wang[1,2], Yayuan Huang [1,3,] *, Xinqi Wang [1,2], Bingyu Li [4], Ming Lei [1,2,] *

[1] College of Environment and Ecology, Hunan Agricultural University, Changsha 410128, China

[2] Hunan Engineering and Technology Research Center for Irrigation Water Purification, Changsha 410128, China

[3] School of Metallurgy and Environment, Central South University, Changsha 410083, China

[4] Institute of Agricultural Environment and Ecology, Hunan Academy of Agricultural Sciences, Changsha 410128, China

* Correspondence: yyhuang@csu.edu.cn; leiming@hunau.edu.cn

- **Field investigation and sample collection**

Table S1. Soil physicochemical properties of sampling sites

sampling site	soil type	pH	Organic matter ($g\cdot kg^{-1}$)	Total N ($g\cdot kg^{-1}$)	Total P ($g\cdot kg^{-1}$)	Total K ($g\cdot kg^{-1}$)	CEC ($cmol\cdot kg^{-1}$)	Total Cd ($mg\cdot kg^{-1}$)
S1	yellow-brown soil	6.12	29.29	0.94	0.22	11.82	13.46	0.14
S2		5.83	34.63	1.15	0.27	10.06	16.23	0.24
S3		5.93	25.45	1.22	0.36	9.83	15.42	0.10
S4		6.09	30.26	1.31	0.31	10.25	15.15	0.28

- **Preliminary Screening of Amendments**

1. Experimental Design and Management

A preliminary field experiment was conducted to screen for the most effective and cost-efficient soil amendments for reducing cadmium (Cd) accumulation in *Lilium*. Four amendments were evaluated: Oyster Shell Powder, Sepiolite, Organic Fertilizer A and Organic Fertilizer B, with their detailed composition and nutritional characteristics provided in Table S2. Oyster shell powder and sepiolite were provided by Hunan Shanshui Qingyang Environmental Protection Technology Co., Ltd (Changsha China). Organic Fertilizer A and Organic Fertilizer B were commercially produced and provided by Hunan Chenhe Eco-agricultural Science and Technology Co., Ltd

(Longhui, China).

The experiment was established in September 2020 at site S4 in Longhui County (Figure 6a). The key physicochemical properties of the experimental soil are presented in Table S1. The tested *Lilium* variety was *Lilium brownii var. viridulum*. The experiment consisted of four amendment treatments, each applied at two rates: a low rate (L) of 4,500 $kg \cdot ha^{-1}$ and a high rate (H) of 9,000 $kg \cdot ha^{-1}$. Each treatment was replicated three times. A control group (CK, no amendment) with three replicates was also included, resulting in a total of 27 experimental plots, each measuring 18 m^2.

The designated amendments were evenly applied to the soil surface as a basal fertilizer and then incorporated into the top 20 cm of the soil layer by plowing. The treated plots were left to equilibrate for 20 days prior to sowing *Lilium* bulbs. All other field management practices, including irrigation and pest control, were consistent with local agricultural standards.

Table S2. Detailed composition and nutritional characteristics of amendments

Amendments	Components	pH	Total Cd ($mg{\cdot}kg^{-1}$)	Organic matter ($g{\cdot}kg^{-1}$)	Total N ($g{\cdot}kg^{-1}$)	Total P ($g{\cdot}kg^{-1}$)	Total K ($g{\cdot}kg^{-1}$)	CaO ($g{\cdot}kg^{-1}$)	SiO_2 ($g{\cdot}kg^{-1}$)	MgO ($g{\cdot}kg^{-1}$)	Al_2O_3 ($g{\cdot}kg^{-1}$)	SO_3 ($g{\cdot}kg^{-1}$)	Fe_2O_3 ($g{\cdot}kg^{-1}$)	TiO_2 ($g{\cdot}kg^{-1}$)
Oyster Shell Powder	Primarily $CaCO_3$	8.73	0.02	N.D.[2]	0.50	21.70	7.40	448.60	242.50	18.60	17.40	11.80	4.10	1.60
Sepiolite	$Mg_8Si_{12}O_{30}(OH)_4(H_2O)_4{\cdot}8H_2O$	9.85	N.D.[1]	N.D.[2]	2.10	9.44	3.70	316.40	512.60	32.20	9.16	6.72	2.19	0.25
Organic Fertilizer A	Fermented livestock manure, rapeseed meal, wheat bran, humic acid, amino acids	7.20	0.40	450.52	15.70	41.00	18.30	N.D.[2]	N.D.[2]	N.D.[2]	N.D.[2]	N.D.[2]	N.D.[2]	N.D.[2]
Organic Fertilizer B	Cow manure	7.50	1.01	520.10	23.60	23.40	15.10	N.D.[2]	N.D.[2]	N.D.[2]	N.D.[2]	N.D.[2]	N.D.[2]	N.D.[2]

[1] N.D. = Not Detected (below the detection limit of the instrument).

[2] N.D. = Not Determined.

2. Sample Collection and Analysis

At the *Lilium* maturity stage in August 2021, bulb samples were collected from each plot using the five-point sampling method. The samples were washed thoroughly with tap water followed by deionized water, then oven-dried at 105°C for 1 hour to deactivate enzymes, and subsequently at 65°C to a constant weight. The dried samples were ground into a fine powder using a stainless-steel grinder and stored in sealed plastic bags for chemical analysis.

Total Cd was extracted by digesting the plant powder in a concentrated HNO_3-$HClO_4$ mixture (4:1, v/v). The Cd concentrations in the final digestion solutions were quantified using an inductively coupled plasma mass spectrometer (ICP-MS, Agilent 7700x, PerkinElmer, Waltham, MA, USA). Analytical quality was assured through the concurrent digestion and analysis of method blanks and a certified plant reference material (GBW10049, GSB-27).

3. Results

Figure S1 shows the effects of four soil amendments on the Cd content in *Lilium* bulbs. In the control group (CK), the mean Cd content in the bulbs was 1.13 ± 0.24 mg·kg^{-1}. The application of all four amendments at both low (L: 4,500 kg·ha^{-1}) and high (H: 9,000 kg·ha^{-1}) rates significantly reduced bulb Cd accumulation compared to the CK. At the low application rate, the Cd content in bulbs was reduced to 0.33 mg·kg^{-1} (Oyster Shell Powder), 0.47 mg·kg^{-1} (Sepiolite), 0.32 mg·kg^{-1} (Organic Fertilizer A) and 0.62 mg·kg^{-1} (Organic Fertilizer B), representing reductions of 70.9%, 58.5%, 71.7% and 45.3%, respectively. At the high application rate, the corresponding Cd contents were 0.27 mg·kg^{-1} (Oyster Shell Powder), 0.28 mg·kg^{-1} (Sepiolite), 0.34 mg·kg^{-1} (Organic Fertilizer A) and 0.64 mg·kg^{-1} (Organic Fertilizer B), with reductions of 76.2%, 74.9%, 69.9% and 43.5%, respectively. Notably, the application of Oyster Shell Powder at the high rate successfully reduced the bulb Cd content to 0.27 mg·kg^{-1}, which is below the limit of 0.30 mg·kg^{-1} set by the "Green Trade Standard for Import and Export of Medicinal Plants and Preparations". Among the amendments, Oyster Shell Powder and Organic Fertilizer A were the most effective. For Oyster Shell Powder, a higher application rate yielded a better result. In contrast, for Organic Fertilizer A, there was no significant difference between the low and high rates, suggesting its high efficiency at the lower dosage. Based on the comprehensive evaluation of Cd immobilization efficiency and cost-effectiveness, Oyster Shell Powder and Organic Fertilizer A at the low application rate (4,500 kg·ha^{-1}) were selected for the subsequent field validation experiment.

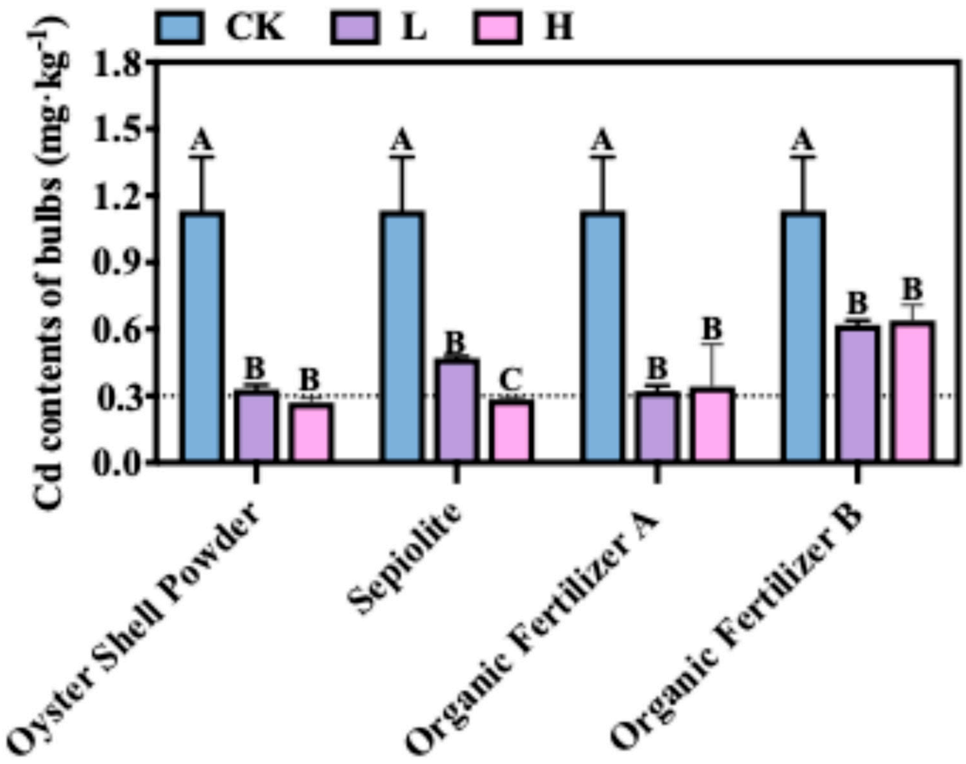

Figure S1. Cd content in bulbs of *Lilium* under different soil amendment treatments. Data are mean SD (n = 3). Different letters indicate significant difference among treatments ($p < 0.05$).
